# Case report: Transmural migration of a gossypiboma with secondary cystolithiasis and urethral obstruction

**DOI:** 10.3389/fvets.2024.1394052

**Published:** 2024-05-15

**Authors:** Meghan L. Lancaster, Chad W. Schmiedt

**Affiliations:** Department of Small Animal Medicine and Surgery, College of Veterinary Medicine, University of Georgia, Athens, GA, United States

**Keywords:** gossypiboma, transmural migration, canine, textiloma, surgical gauze, urinary obstruction, cystolithiasis

## Abstract

This report describes a case of transmural migration of a gossypiboma from the peritoneum into the urinary bladder in a 4-year-old, female spayed, mixed-breed dog. The dog was presented on an emergency basis for complete urethral obstruction with radiographic evidence of urocystolithiasis. An exploratory laparotomy was performed and a 4–5 cm mass was identified which was confluent with the apex of the urinary bladder. The mass and bladder were exteriorized and isolated, and an apical, partial cystectomy was performed to remove the mass and gain access to the uroliths within the lumen of the bladder. A 4×4 surgical sponge was identified within the trigone of the bladder, it had multiple uroliths; another sponge was also found within the mass itself. This case demonstrates an atypical cause of urethral obstruction and serves as the first reported case of transmural migration of a gossypiboma into the urinary bladder of a dog. It also illustrates the importance of establishing routine operating procedures including gauze counts and the use of radiopaque-labeled surgical gauze.

## 1 Introduction

Gossypiboma is a rarely reported condition in veterinary medicine in which surgical gauze is retained within the patient following completion of the surgical procedure. It is a condition that can be challenging to diagnose because of variable and often nonspecific clinical signs, prolonged time before presentation of complications, and occasionally incomplete medical records. When reported in small animals, gossypibomas are most often located within the abdomen and associated with a prior ovariohysterectomy (1, 2). While the exact incidence of gossypibomas is unknown in both human and veterinary medicine (2, 3), one study in human medicine identified several risk factors for their occurrence, including emergency surgeries, increased body-mass index, and unexpected changes in the procedure that had to be performed (3). In that study, it was found that the likelihood of a retained foreign body was nine times more likely in emergency cases, four times more likely in cases where the initial surgical plan deviated from the final operation performed, and had a cumulatively increasing risk with increasing body-mass index (3).

This case report describes the clinical presentation, treatment, and outcome of an abdominal gossypiboma, presumably left in the dog’s abdomen during a previous ovariohysterectomy surgery. It had migrated from the peritoneal cavity through the wall of the urinary bladder and led to difficulty in passing urine, and eventually urinary tract obstruction in the dog.

## 2 Case description

### 2.1 Clinical history

A 5-year-old, female spayed, 24.8 kg mixed breed dog was presented with a 1-day history of stranguria, panting, vocalization, and emesis with no observed urination. About two weeks before presentation, an episode of hematuria was noted by the owner, but no other abnormalities were noted. The dog had no previous medical concerns and the only surgical history was a routine ovariohysterectomy performed by the primary care veterinarian several years ago and shortly after adoption.

The dog was initially brought to her local emergency veterinarian where it received supportive treatment, including an antiemetic, analgesia, IV fluids, and antibiotics. A cystocentesis was performed to withdraw150 ml of dark, hemorrhagic urine, from which a sample was collected. Urinalysis revealed hematuria, pyuria, and bacteriuria. Blood was submitted for a complete blood count and serum biochemical profile. The dog was azotemic with an elevated creatinine (1.9 mg/dL; range, 0.5–1.8 mg/dL) and had mild hyperglobulinemia (4.9 g/dL; range, 2.5–4.5 mg/dL). The dog was also noted to be anemic (32.8%; range, 37.3–61.7%) and thrombocytopenic (118 × 10^3^/μL; range, 148–484 × 10^3^/μL). The total white blood cells were normal with mild lymphopenia (0.88 × 10^3^/μL; range, 1.05–5.10 × 10^3^/μL) and suspected band cells.

At the emergency clinic, the dog was then sedated for abdominal radiographs and for fitting an indwelling urinary catheter. The dog was hospitalized overnight for continued monitoring and treatment and then referred for further care.

### 2.2 Physical exam

Upon presentation to the University of Georgia, Veterinary Teaching Hospital the following day, the dog was quiet, alert, and responsive with normal vital parameters. The previous phrasing conveys slightly more information as to the functionality of the catheters and the decision to not replace them, however, this can also be inferred by the current phrasing so we find this to be an acceptable edit. The remainder of the physical examination was within normal limits. A triage abdominal ultrasound was performed which showed no free peritoneal fluid. However, during this evaluation, a round structure with a hyperechoic, spiculated center was observed within the urinary bladder.

### 2.3 Diagnostic findings

**Figure 1:**
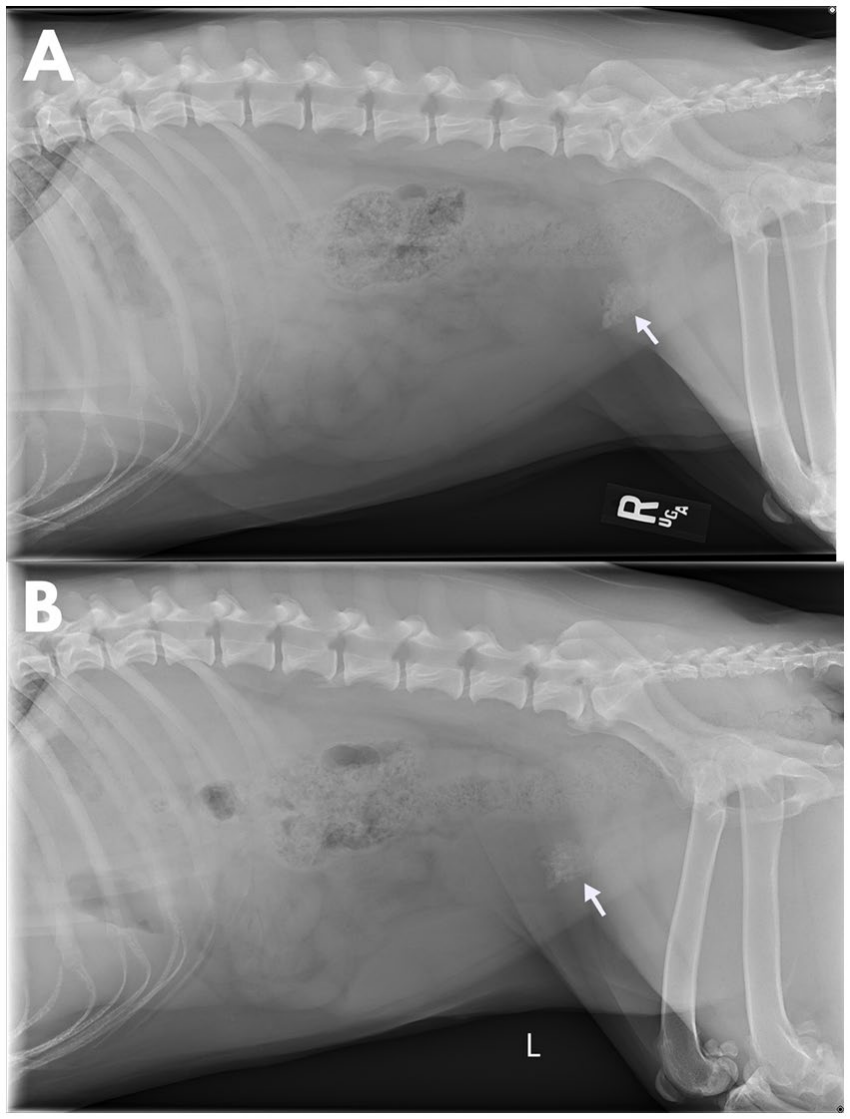
Lateral abdominal radiographs obtained post-placement of an indwelling urinary catheter in a 5-year-old, spayed female, mixed breed dog that was evaluated for a 2-week history of lower urinary signs which progressed to complete urethral obstruction. There is an aggregation of numerous, small, irregularly marginated, mineral opacities at the neck of the bladder (arrow). These opacities do not shift between right **(A)** and left **(B)** lateral recumbency illustrating a lack of gravity dependence typically observed in urocystoliths.

Radiographs performed by the referring emergency service were reviewed. Radiopaque urocystoliths were located near the trigone of the bladder with an atypical, non-gravity-dependent distribution (Figure 1). Repeat urine analysis revealed elevated protein (4+), elevated pH (8.5), hematuria (<10 RBC/HPF), pyuria (5–10 WBC/HPF), triple phosphate crystals (few), and bacteriuria (many). Repeat blood work confirmed a mild anemia (40.4%; range, 42.2–59.8%). Complete blood count showed a leukocytosis (30.8 × 10^3^/μL; range, 4.2–12.9 × 10^3^/μL) characterized by a mature neutrophilia (24.034 × 10^3^/μL; range, 2.700–8.500 × 10^3^/μL), monocytosis (1.232 × 10^3^/μL; range, 0.100–1.000 × 10^3^/μL), and eosinophilia (4.928 × 10^3^/μL; range, 0.00–1.200 × 10^3^/μL), consistent with a chronic inflammatory process.

Based on the diagnostic results and consistent clinical history, the dog was diagnosed with a lower urinary tract obstruction and urinary tract infection secondary to urocystolithiasis, and surgical removal of the cystoliths was suggested.

### 2.4 Treatment and outcomes

The patient was premedicated with hydromorphone (0.1 mg/kg IV) and dexmedetomidine (1 μg/kg IV) and anesthesia was induced with ketamine (2 mg/kg IV) and propofol (4.8 mg/kg IV) titrated to effect. A surgical plane of anesthesia was maintained with isoflurane (1–2%) and lidocaine (2 mg/kg IV bolus followed by a 3 mg/kg/h CRI). The patient was positioned in dorsal recumbency and prepared for aseptic surgery. An approximately 8 cm ventral midline incision was made in the caudal abdomen to facilitate a routine cystotomy. Upon opening the abdomen, multiple adhesions were observed. The urinary bladder was identified and a large (4–5 cm), round, tan, firm mass was identified, which was firmly adherent to the apex of the bladder with multiple omental adhesions. The adhesions were dissected and the bladder and associated mass were exteriorized and isolated.

**Figure 2:**
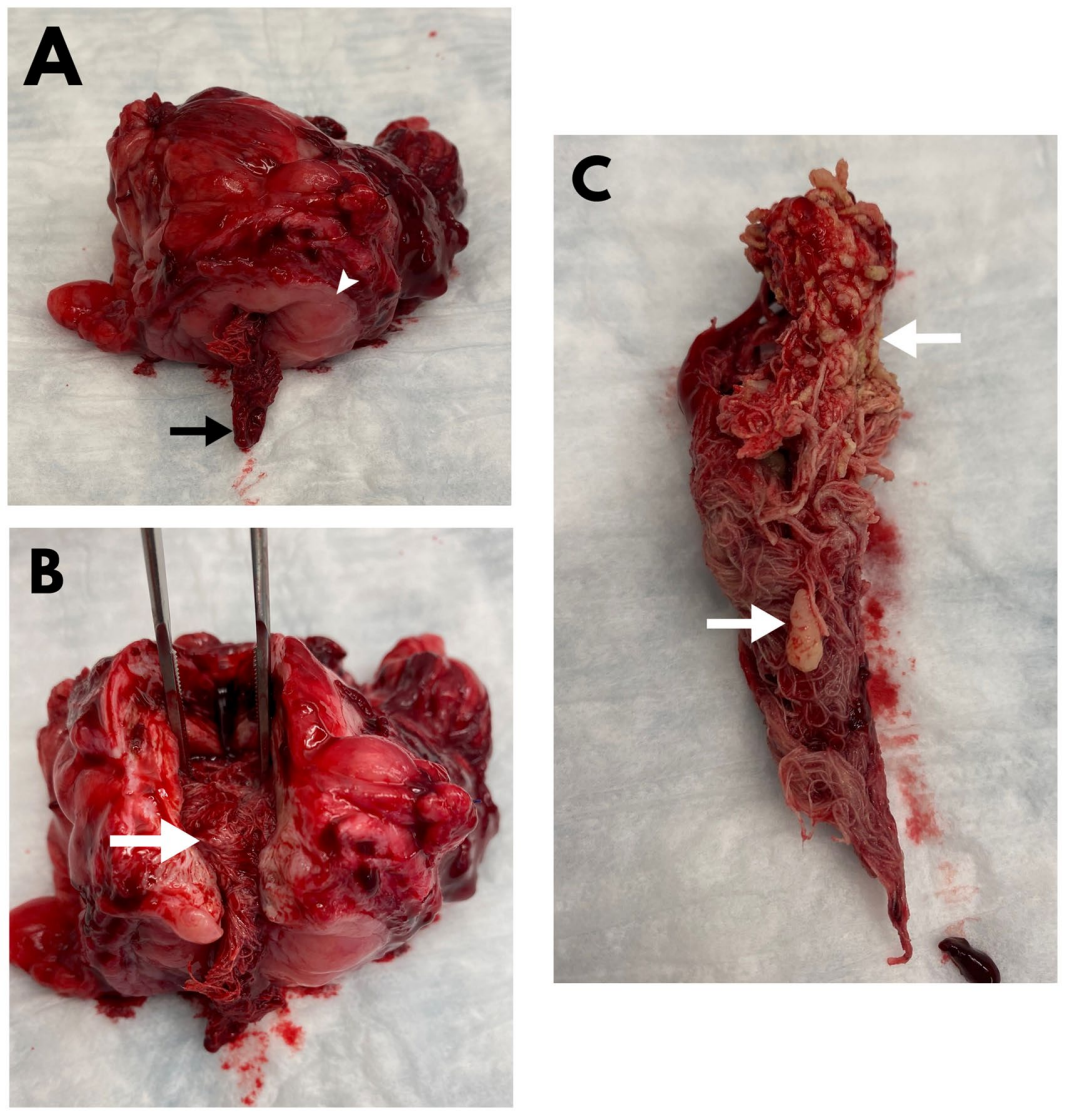
Postoperative images of a mass removed from the apex of the urinary bladder **(A,B)** alongside a urolith-laden surgical gauze removed from within the lumen of the urinary bladder **(C)** of the dog described in Figure 1. **(A)** A surgical gauze (arrow) is shown protruding from a defect on the mucosal surface (arrowhead) of the bladder mass. **(B)** An incision through the luminal defect along the long aspect of the bladder mass shows the encapsulated surgical gauze (arrow) within the gossypiboma. **(C)** The additional gauze within the bladder lumen was retrieved from the neck of the bladder and is shown to have numerous uroliths (arrows) adhered to its surface.

An apical partial cystectomy was performed to remove the mass and facilitate the removal of the cystoliths. When viewed from the lumen of the bladder, at the junction of the mass and the bladder, an opening was seen in the mass and a 4×4 surgical gauze was present inside the mass (Figure 2). The remainder of the mass was submitted for histopathologic evaluation which revealed the mass to be a bladder wall with evidence of severe, chronic inflammation resulting in a marked expansion of the submucosa by collagen and with infiltration of macrophages, lymphocytes, and plasma cells. The mucosa was multifocally ulcerated with diffuse squamous metaplasia. No cloth-like or suture material was present within the bladder wall on microscopic examination.

A second 4×4 gauze was identified and extracted from within the lumen of the urinary bladder at the trigone. There were multiple cystoliths incorporated within the gauze, from which a sample was submitted for stone analysis and identified to be primarily struvite in nature. A swab of the bladder mucosa was collected for culture, which was later identified as *Staphylococcus pseudintermedius*. Based on sensitivity data, the dog was discharged on an additional 10-day course of amoxicillin/clavulanate (Clavamox, 14.8 mg/kg PO q12).

Subsequent to the removal of the surgical gauze, the bladder was emptied of any remaining stones, and an 8Fr red rubber catheter was easily passed normograde and then again retrograde with repeated flushing of the urethra and urinary bladder to ensure the complete removal of any other small stones. The bladder was closed as per routine procedure and a gauze count was confirmed. The remainder of the abdominal assessment was unremarkable and the abdomen was closed as per routine procedure. Post-operative radiographs were performed before recovery to confirm that no residual stones were present. The dog recovered from surgery without complications and was discharged after two days of post-operative hospitalization. The dog was able to urinate normally after discharge.

## 3 Discussion

Transmural migration of a gossypiboma has been documented in human medicine, with passage of the gauze in feces following migration into the colon, lower urinary signs secondary to migration into the urinary bladder, and even migration across the diaphragm resulting in invagination into the lung parenchyma (4). This is the first case report documenting the transmural migration of an abdominal gossypiboma into the bladder of a dog, and one of only a small number reporting the phenomenon of transmural migration in animals (1, 5, 6). Before this report, transmural migration has only been reported in dogs in association with the gastrointestinal tract, which is also the most common site of migration in humans (1, 4).

A systematic review of human cases of transmural surgical sponge migration reported migration into the bladder in 7 of 64 cases, with all 7 patients presenting urinary tract infections and obstruction (4). One veterinary case documented adherence of the gossypiboma to the bladder wall resulting in gross edematous and erythematous changes to the bladder and histopathologic changes consistent with chronic cystitis. Although the granuloma in that case was distinct and could be dissected off the bladder without a partial cystectomy (7), the observed inflammatory changes progressing into adjacent organs could have represented an early stage of transmural migration.

Diagnosis of a retained gauze is often achieved preoperatively with the use of various imaging modalities and is made easier with the use of radiopaque markers. In the absence of these markers, the imaging findings can be subtle, with top differentials of inflammatory lesions and neoplasia (8). The most commonly reported radiographic features are mass effects, a gas pattern within a mass effect that has been described in a whirl or speckled pattern, peripheral calcification of the lesion, and evidence of an intestinal obstruction (8). Notably, these described gas changes were absent within this case, and it is presumed that this may have been a factor of the migration into the urinary bladder, immersion of the sponge in urine, and the prolonged time-course of this patient’s disease. The gauze that fully migrated into the urinary bladder did display calcification, however, serving as a nidus of stone formation. This also resulted in the atypical, non-gravity-dependent distribution of the cystoliths on the abdominal radiographs.

Radiopaque-labeled surgical gauzes are superior to routine sterile gauze on several counts and should be considered the standard of care for veterinary medicine. While radiographic changes can be seen even without the radiopaque marker, they are more subtle, as previously stated, and are not always present, such as in the case of this patient. Furthermore, unlabeled gauze is commonly purchased in bulk and then separately packaged by hospital staff for sterile surgery, but in doing so, the exact number in each sterile pack may not be consistent. Radiopaque labeled gauze is typically purchased from the manufacturer in pre-sterilized packs of consistent and predictable quantities, making it much easier to rapidly confirm the number of gauze on the table. The marker can also be used to confirm that no surgical gauze was left behind in instances where a complete gauze count cannot be confirmed by hand counting at the end of the surgical procedure. This case illustrates the need for standardization of operating room procedures in veterinary practices with the use of radiopaque-labeled surgical gauze, pre-and post-procedure counts, operating room safety checklists, and complete documentation of surgical reports. A well-documented surgical history and standardized operating room protocols may allow for earlier identification of a gossypiboma as a differential or even recognition of the presence of gauze within the surgical site before closure.

## Data availability statement

The original contributions presented in the study are included in the article/supplementary material, further inquiries can be directed to the corresponding author.

## Author contributions

ML: Investigation, Writing – original draft, Writing – review & editing. CS: Conceptualization, Investigation, Supervision, Writing – review & editing.
